# Neonatal Anemia in Preterm Infants: Are We Overtransfusing?

**DOI:** 10.7759/cureus.87657

**Published:** 2025-07-10

**Authors:** Pravati Jena, Rojalin Rout, Rama K Gudu, Deepti D Pradhan, Santosh K Panda

**Affiliations:** 1 Pediatrics, Kalinga Institute of Medical Sciences, Bhubaneswar, IND; 2 Neonatology, Kalinga Institute of Medical Sciences, Bhubaneswar, IND

**Keywords:** anemia, blood transfusion, hemoglobin, neonate, neonatology, packed red blood cell transfusion, preterm, red blood cell

## Abstract

Aims and objectives: The purpose of this study was to ascertain the appropriate packed red blood cell transfusion (PRBCT) practice for preterm infants consistent with multiple international guidelines.

Method: This retrospective study was conducted at a tertiary care center in India from February 2019 to December 2020. All preterm neonates born at <32 weeks of gestation who received PRBCTs during the study period were included. The hemoglobin (Hb) levels at the time of transfusion were analyzed to assess adherence to the transfusion thresholds recommended by the British Committee for Standards in Haematology (BCSH), Canadian Blood Services (CBS), and the Australian National Blood Authority (ANBA), considering postnatal age and the need for respiratory support.

Results: A total of 43 preterm neonates (gestational age, 28.08±1.08 weeks; birth weight, 1,018±0.027 g) received 78 PRBCTs. Specifically, nine (11.5%) PRBCTs were given in the first week, 14 (18%) in the second week, and 55 (70.5%) at three or more weeks of age. Further, nine (11.5%) PRBCTs were given during mechanical ventilation (MV), 49 (63%) with non-invasive ventilation (NIV) or oxygen (O₂) support, and 20 (25.5%) without O₂ support. The appropriateness of all of the PRBCTs per the BCSH, CBS, and ANBA guidelines was 63/78 (80.7%), 58/78 (74.3%), and 78/78 (100%), respectively. Most of the neonates on respiratory support received PRBCTs in a manner consistent with the CBS, BCSH, and ANBA guidelines, whereas most of the neonates not on oxygen (O₂) therapy received PRBCTs for late anemia (at three weeks or more of age) at a higher Hb level than that suggested by the CBS and BCSH guidelines but consistent with the ANBA guidelines.

Conclusion: In this transfusion audit study, liberal PRBCT practice was observed for late anemia among stable preterm infants, consistent with the CBS and BCSH blood transfusion guidelines. Hence, there is room for improvement in PRBCT policies in terms of quality-improvement interventions to prevent over-transfusion.

## Introduction

Preterm neonates are at increased risk for anemia, and packed red blood cell transfusion (PRBCT) is a common therapeutic intervention for the management of this condition [1]. Low gestational age, low birth weight, and the presence of cardio-respiratory morbidity in preterm infants increase the need for PRBCTs. Transfusions increase oxygen (O₂) delivery to tissues, help manage hypoxemia and apnea, and promote weight gain, but they simultaneously increase the risk of multiple complications.

Hemoglobin (Hb) is the most common indicator for PRBCT. The decision to transfuse is primarily based on Hb thresholds, which vary according to the severity of respiratory complications. There is wide variation in the recommended threshold across neonatal units worldwide [2]. Most neonatal units adopt one of the international guidelines, namely the Canadian Blood Services (CBS), the British Committee for Standards in Haematology (BCSH), or the Australian National Blood Authority (ANBA). All guidelines recommend the Hb threshold for PRBCT based on chronological age and the need for respiratory support [3-5]. Therefore, this study aims to determine whether our PRBCT practice for preterm infants was consistent with these global transfusion guidelines.

## Materials and methods

This retrospective study was conducted in a tertiary center in eastern India from February 2019 to December 2020 with permission from the Institutional Ethics Committee, Kalinga Institute of Medical Sciences (Bhubaneswar, OR, IND). Because of the study design, individual patient consent was not obtained. All preterm neonates under 32 weeks who received PRBCTs in the neonatal intensive care unit during the study period were included. Neonates receiving transfusions during surgical procedures, in the immediate postoperative period, or undergoing repeated transfusions within 24 hours were excluded, as transfusion decisions in these contexts are often driven by acute clinical indications rather than standard guideline thresholds.

We entered the detailed demographic characteristics of the enrolled neonates and the data associated with each transfusion episode in a predefined pro forma. These characteristics and data included the Hb level (g/dl) prior to transfusion, chronological age (week of age), and the type of respiratory support, whether mechanical ventilation (MV), non-invasive ventilation (NIV) such as noninvasive positive pressure ventilation (NIPPV), continuous positive airway pressure (CPAP), high flow, or with or without O₂ therapy during the PRBCT. The suggested Hb (g/dl) threshold for PRBCTs under the BCSH, ANBA, and CBS guidelines is listed in Table 1. Note: In certain clinical situations, the Hb threshold may increase to 8.5 g/dl for neonates without O2 under the BCSH guidelines.

**Table 1:** The Hb (g/dl) threshold for PRBCTs per the BCSH, ANBA, and CBS guidelines PRBCT: Packed red blood cell transfusion, BCSH: British Committee for Standards in Haematology, ANBA: Australian National Blood Authority, CBS: Canadian Blood Services, NIV: Noninvasive ventilation, MV: Mechanical ventilation, CPAP: Continuous positive airway pressure, O_2_: Oxygen

Guideline	Category	Hb (g/dl) threshold	Timeline
BCSH	Neonates with MV	<12	24 hours
		<12	1 week
		<10	2 weeks
		<10	≥ 3 weeks
	Neonates with NIV/O2	<12	24 hours
		<10	1 week
		<9.5	2 weeks
		<8.5	≥ 3 weeks
	Neonates without O2	<10	24 hours
		<10	1 week
		<7.5	2 weeks
		<7.5	≥3 weeks
ANBA	Neonates with respiratory support (MV/CPAP/high-flow/O_2_)	11-13	1 week
		10-12.5	2 weeks
		8.5-11	≥3 weeks
	Neonates without respiratory support	10-12	1 week
		8.5-11	2 weeks
		7-10	≥3 weeks
CBS	Neonates with respiratory support	<11.5	1 week
		<10	2 weeks
		<8.5	≥3 weeks
	Neonates without respiratory support	10	1 week
		8.5	2 weeks
		7.5	≥3 weeks

Assuming a 30% prevalence of inappropriate PRBC transfusions based on a previous study [6] and using a 10% margin of error at a 95% confidence level, the estimated sample size was 81. Our audit included 78 transfusion episodes, which is close to the calculated requirement and offers adequate power for descriptive analysis. In the analysis of the data, the average of the quantitative variables was expressed as the mean (SD), and the qualitative variable was expressed as the frequency (%). The primary outcome was the appropriateness of the PRBCTs according to the various international transfusion guidelines expressed as a proportion. The descriptive statistics for the data analysis were calculated using the DATAtab statistical calculator (DATAtab Seiersberg, STE, AUT).

## Results

During the study period, 43 (29%) of the 148 admitted preterm neonates received a total of 78 PRBCTs. The mean gestational age and birth weight of these neonates were 28.08±1.08 weeks and 1018±0.027 g, respectively. Of these neonates, 28 (65.2%) were male, 16 (37.2%) were delivered by cesarean, and 21 (49%) were of extremely low birth weight (ELBW) (Table 2).

**Table 2:** Demographic characteristics of preterm neonates who received PRBCTs ^#^Mean (SD), rest frequency (%); ELBW: Extremely low birth weight, PRBCT: Packed red blood cell transfusion

Neonatal characteristics	Frequency (%) and mean (SD)
Gestational age (weeks)^#^	28.08 (1.08)
Birth weight (grams)^#^	1018 (0.027)
Gender (male)	28 (65.2%)
Gender (female)	15 (34.8%)
Mode of delivery (caesarean)	16 (37.2%)
Number of ELBW	21 (49%)

A total of 22 (51.2%) neonates received a single PRBCT, 14 (30.7%) neonates received two PBRCTs, and seven (16.1%) neonates received three or more PRBCTs. A total of 21 (80.7%) of 26 ELBW neonates (birth weight <1000 g) and 22 (18%) of 122 very low birth weight (VLBW) neonates (1000-1500 g) received PRBCTs during their hospital stays.

The proportion of transfusions received during the first week, second week, and subsequent weeks after the birth of the neonates was nine (11.5%), 14 (18%), and 55 (70.5%), respectively. Of 78 transfusion episodes, nine (11.5%) were administered during MV, 49 (63%) during NIV and O₂ support, and 20 (25.5%) without O₂ support (Table 3). Of the 20 neonates transfused PRBCs without O₂, 14 (70%) were asymptomatic and at risk of loss to follow-up, one (5%) continued to fail to thrive, three (15%) had isolated tachycardia, and two (10%) had recurrent apnea. Six of the above clinical scenarios were resolved with PRBCTs.

**Table 3:** Age of neonates and need for respiratory support during PRBCT in preterm neonates PRBCT: Packed red blood cell transfusion

Age of neonates during transfusion	Frequency (%)
Transfusions received during the first week	9 (11.5%)
Transfusions received during the second week	14 (18%)
Transfusions received ≥ 3 weeks	55 (70.5%)
Need for respiratory support during transfusion	Frequency (%)
Transfusion during mechanical ventilation	9 (11.5%)
Transfusion during non-invasive ventilation and O₂ support	49 (63%)
Transfusion without O₂ support	20 (25.5%)

All of the neonates received PRBCTs during MV in a manner consistent with the CBS, BCSH, and ANBA transfusion guidelines. Of the PBRCTs that were given to the neonates receiving NIV or O₂ support, 48/49 (98%) followed the BCSH guidelines, and all 49 (100%) followed both the CBS and ANBA guidelines. Of the transfusions without O₂ support, six (30%) were consistent with the BCSH guidelines, none followed the CBS guidelines, and all were consistent with the ANBA guidelines (Table 4). Overall, the PRBCTs were consistent with the BCSH, CBS, and ANBA guidelines in 63/78 (80.7%), 58/78 (74.3%), and 100% of the cases, respectively.

**Table 4:** Adherence to the Hb threshold for PBRCT with the BCSH, ANBA, and CBS guidelines Hb: Hemoglobin, PRBCT: Packed red blood cell transfusion; MV: mechanical ventilation; NIPPV: Non-invasive positive pressure ventilation; O_2_: Oxygen, CPAP: Continuous positive airway pressure, BCSH: British Committee for Standards in Haematology, ANBA: Australian National Blood Authority, CBS: Canadian Blood Services a/b: a is the number of transfusions adhering to the guidelines, and b is the total number of transfusions; *Mechanical ventilation, CPAP, high flow, O_2_

Postnatal PRBCT (n=78)	BCSH			ANBA		CBS	
a/b (%)	MV (n=9)	NIPPV, O_2_ (n=49)	No O2 (n=20)	Respiratory support* (n=58)	No respiratory support (n=20)	Respiratory support* (n=58)	No respiratory support (n=20)
1st week (n=9)	7/7 (100%)	2/2 (100%)	-	9/9 (100%)	-	9/9 (100%)	-
2nd week (n=14)	1/1 (100%)	12/13 (92.3%)	-	14/14 (100%)	-	14/14 (100%)	-
≥ 3rd week (n=55)	1/1 (100%)	34/34 (100%)	6/20 (30%)	35/35 (100%)	20/20 (100%)	35/35 (100%)	0/20 (0%)
Overall	9/9 (100%)	48/49 (98%)	6/20 (30%)	58/58 (100%)	20/20 (100%)	58/58 (100%)	0/20 (0%)

## Discussion

This is the first monocentric study from a low- to middle-income country designed to report whether the PRBCT practice for preterm infants is consistent with various international guidelines. The neonates with severe to moderate respiratory impairment received PRBCTs in a manner consistent with the ANBA, BCSH, and CBS guidelines, whereas most stable preterm infants with late anemia received PRBCTs liberally, consistent with the CBS and BCSH guidelines.

The need for PRBCTs among the ELBW and VLBW neonates in our study was similar to that in many previous studies [7,8]. The landmark Premature Infant in Need of Transfusion (PINT) trial [9] and the findings of Chen et al. [10] have convinced neonatologists to transfuse at lower Hb thresholds by indicating a lesser requirement for PRBCTs without compromising any neonatal morbidities, i.e., severe intraventricular hemorrhage, retinopathy of prematurity, or chronic lung disease. Subsequently, the Transfusion of Premature (TOP) trial [11] and the Effects of Transfusion Thresholds on Neurocognitive Outcomes of Extremely Low-Birth-Weight Infants (ETTNO) group [12] found no reduction in death or disability with liberal PRBCTs and advocated for restrictive strategies. The fact that the Hb threshold suggested by the BCSH and the CBS is consistent with the restrictive PRBCT strategy, whereas the Hb threshold remained in the range of the restrictive to liberal Hb cut-off in the ANBA guidelines, explains the greater consistency of our PRBCT practice with the ANBA guidelines [9].

Packed red blood cell transfusions in moderate-to-severe cardiorespiratory cases in preterm infants can be lifesaving, and transfusions in stable preterm infants are required in the context of anemia of prematurity with failure to thrive, heart failure, and tissue hypoxia. Considering the dropout in the post-discharge follow-up, the lack of serial Hb estimation, and the home monitoring of clinical signs and symptoms of severe anemia similar to the situation in most low- and middle-income countries, we conducted PRBCTs liberally in stable preterm infants after one-on-one parental discussions. By contrast, an Indian study found that 30% of the PRBCTs in the neonatal intensive care unit (NICU) were inconsistent with the BCSH guidelines, though most involved sick neonates [6].

We acknowledge that this study is subject to significant limitations. First, the data were analyzed retrospectively and from a single center, and the sample size was small. Second, we routinely evaluated the reticulocyte percentage as a marker of erythropoiesis, coupled with the Hb level, for anemia screening among growing preterm infants; however, the usefulness of this practice is beyond the scope of this study. Again, the short- and long-term outcomes of neonates who received liberal transfusions have not been reported. 

## Conclusions

We observed that many stable preterm neonates were transfused liberally with PRBCs in our clinical practice compared with the internationally published guidelines. This discrepancy raises concerns about adherence to best practices and the potential adverse effects for preterm infants. Further investigation into the criteria for transfusion in this population is necessary to align clinical practice with established guidelines. Hence, this study highlights the importance of transfusion audits in neonatal units and provides a basis for future quality improvement measures in PRBCT strategy, considering Hb or hematocrit threshold and reticulocyte count parameters to avoid over-transfusion in stable preterm neonates.
